# Supplementary material for: Reprogramming of the antimycin NRPS-PKS assembly lines inspired by gene evolution
Source: Nat Commun. 2018 Aug 30;9:3534. doi: 10.1038/s41467-018-05877-z (PMC6117356; doi:10.1038/s41467-018-05877-z)
Supplement: Supplementary file 1 — Supplementary Information [file 41467_2018_5877_MOESM1_ESM.docx]

## **Reprogramming of the antimycin NRPS-PKS assembly lines inspired by gene evolution**

Awakawa *et al.*

**Supplementary Note 1. Construction of pKU518J06 and pKU518nant**

Genomic libraries were constructed containing ~200 kbp insert of producing strains, Streptomyces sp. ML55 and Streptomyces orinoci NBRC13466, respectively using the integrating vector, pKU518. Detailed method for construction of the BAC library was previously described (M. Komatsu, K. Komatsu, H. Koiwai, Y. Yamada, I. Kozone, M. Izumikawa, J. Hashimoto, M. Takagi, S. Omura, K. Shin-ya, D. E. Cane, H. Ikeda, ACS Synth. Biol. 2013, 2, 384-396; M. Komatsu, T. Uchiyama, S. Ōmura, D. E. Cane, H. Ikeda, Proc. Natl. Acad. Sci. USA 2010, 107, 2646-2651.). Clones carrying the entire biosynthetic gene clusters for targeted compounds were screened by PCR amplification. As a result, pKU518J06P3-P19 (with 177 kbp of insert for JBIR-06 production, signated as pKU518J06) and pKU518nantP1-P12 (181 kbp for neoantimycin production, signated as pKU518nant), respectively were efficiently obtained. Primer sequence and constructed vector were summarized in **Supplementary Table 1** **and** **2**, respectively.

**Supplementary Note 2. Construction of pKU518nantΔ*natD*::*smlCTE***

We prepared Fragment 1 (858 bp) encoding SmlC-TE domain, amplified with the primers I and II, and pKU518J06 as a template, Fragment 2 (958 bp) containing AprR, amplified with the primers III and IV, and pIJ773 as a template, Fragment 3 (304 bp) containing ermE promoter, amplified with the primers V and VI, and pTYM19ep (gift from Prof. Onaka) as a template. Fragment 1-3 was integrated into pUC19 cut by EcoRI and HindIII with in-fusion to yield pUC19-SmlCTE-AprR-Perm. RED Fragment SmlCTEAprPerm was amplified with the primers VII and VIII, and pUC19-SmlCTE-AprR-Perm as a template, and integrated into pKU518nant in NEB10β harboring pKU518nant and pKD78 to give pKU518nantΔ*natD::smlCTE.* The underlined letters in primers represent the nucleotide extensions used for in-fusion (Takara), the italic letters in primers represent the nucleotide extensions used for Red/ET recombination, and the experiment was done according to the protocol from John Innes Centre.

**Supplementary Note 3. Construction of pKU518J06ΔsmlCTE**

Fragment 1 (168 bp) encoding C-terminus of NatC, amplified with the primers IX and X, and pKU518nant as a template, fragment 2 (882 bp) including AprR, amplified with the primers XI and XII, and pIJ773 as a template, and fragment 3 (258 bp) including PermE, amplified with the primers XIII and XIV, and pTYM19ep as a template. Fragment 1-3 was integrated into pUC19 cut by EcoRI and NdeI with in-fusion to yield pUC19-CtNatCAprRPerm. RED Fragment CtNatCAprPerm was amplified with the primers XV and XVI, and pUC19-CtNatCAprRPerm as a template, and integrated into pKU518J06 to give pKU518J06Δ*smlCTE* through Red/ET.

**Supplementary Note 4. Construction of pZH2-NatD**

The DNA sequence encoding NatDE was amplified with the primers XVII and XVIII, and pUC19-NatDfixed as a template, and integrated into the downstream of *ermE* promoter in pZH2, a φC31 integration vector, consisted of pUC19, AprR (from pIJ773), φC31 and PermE (from pTYM19ep).

pUC19-NatDfixed was constructed through connecting six PCR fragments, 1, 2, 3 (consisted of 3-1, 3-2, 3-3, 3-4, and 3-5), 4, 5-NatE, and AprR-PermE through in-fusion with adding nonsense mutation in the junction between each fragment (**Supplementary Fig. 51**). Firstly, Fragment 1~2, 3-1~3-2, 3-3~3-5, and 5+NatE+AprR+PermE were connected and subcloned into EcoRI and HindIII site of pUC19 with in-fusion, and fragment 4 was purchased from Greiner. Next, Fragments 1-2, 3, 4, and 5+NatE+AprR+PermE were connected and cloned into EcoRI and HindIII site of pUC19 with in-fusion. The primer sequences were listed in **Supplementary Table 1**.

**Supplementary Note 5. Construction of pZH2-SmlCAT_ant_-AntEV350G**

The fragment 1 was amplified with the primers XXI and XXII, DNA with underline was used for in-fusion and encoded 5AA mutated in this study), and the fragment 2 was amplified with the primers XXIII and XXIV, and pKU518J06 as a template, and integrated into pZH02 to give pZH2-SmlCAT_ant_ by in-fusion. The fragment containing *PermE-antEV350G* was amplified with the primers XXV and XXVI, and integrated into the HindIII site of pZH2-SmlCAT_ant_ by in-fusion, to give pZH2-SmlCAT_ant_-AntEV350G.

**Supplementary Note 6. Construction of pKU518J06ΔsmlC**

Fragment (1298 bp) encoding AprR and PermE was amplified with the primers XIX and XX, and pUC19-CtNatCAprRPerm as a template, and integrated into pKU518J06 to give pKU518J06**Δ**smlC through RED/ET.

| Gene | Amino acid  (base pairs) | Protein homologue  [strain] (Accesion number) | Identity /Positive  (%) | Proposed function |
| --- | --- | --- | --- | --- |
| *orf-1* | 454 (1365) | hypothetical protein [Frankia sp. G2]  (WP_091287320) | 29/45 | Hypothetical protein |
| *smlA* | 172 ([519](http://biosyn.nih.go.jp/2ndfind/show.cgi?type=nuc&id=151850670916787.2)) | Ant2A [Streptomyces blastmyceticus] ([BAM21045](https://www.ncbi.nlm.nih.gov/nuccore/1173163)) | 83/91 | sigma factor |
| *smlB* | [4586](http://biosyn.nih.go.jp/2ndfind/show.cgi?type=ami&id=151850670916787.3) ([13761](http://biosyn.nih.go.jp/2ndfind/show.cgi?type=nuc&id=151850670916787.3)) | Ant2C [Streptomyces blastmyceticus] (BAM21047) | 58/67 | NRPS (C-A-T,C-A-KR-T,C-A-KR-T) |
| *smlC* | [1650](http://biosyn.nih.go.jp/2ndfind/show.cgi?type=ami&id=151850670916787.4) ([4953](http://biosyn.nih.go.jp/2ndfind/show.cgi?type=nuc&id=151850670916787.4)) | Hypothetical protein ACZ90_45135 [Streptomyces albus subsp. albus] (KUJ65684) | 62/71 | PKS  (KS-AT-MT-T-TE) |
| *smlD* | [264](http://biosyn.nih.go.jp/2ndfind/show.cgi?type=ami&id=151850670916787.5) ([795](http://biosyn.nih.go.jp/2ndfind/show.cgi?type=nuc&id=151850670916787.5)) | thioesterase [Streptomyces cattleya]  ([WP_078590533](https://www.ncbi.nlm.nih.gov/nuccore/121652)) | 57/69 | thioesterase |
| *smlE* | [214](http://biosyn.nih.go.jp/2ndfind/show.cgi?type=ami&id=151850670916787.6) ([645](http://biosyn.nih.go.jp/2ndfind/show.cgi?type=nuc&id=151850670916787.6)) | AntQ [Streptomyces blastmyceticus] ([AGG37767](https://www.ncbi.nlm.nih.gov/nuccore/1173163)) | 58/62 | phosphopantetheinyl transferase |
| *smlF* | [492](http://biosyn.nih.go.jp/2ndfind/show.cgi?type=ami&id=151850670916787.7) ([1479](http://biosyn.nih.go.jp/2ndfind/show.cgi?type=nuc&id=151850670916787.7)) | Ant2F [Streptomyces blastmyceticus] ([BAM21051](https://www.ncbi.nlm.nih.gov/nuccore/1173163)) | 81/87 | AMP-ligase |
| *smlG* | [81](http://biosyn.nih.go.jp/2ndfind/show.cgi?type=ami&id=151850670916787.8) ([246](http://biosyn.nih.go.jp/2ndfind/show.cgi?type=nuc&id=151850670916787.8)) | Ant2G [Streptomyces blastmyceticus] (BAM21052) | 88/94 | Carrier protein |
| *smlH* | [334](http://biosyn.nih.go.jp/2ndfind/show.cgi?type=ami&id=151850670916787.9) ([1005](http://biosyn.nih.go.jp/2ndfind/show.cgi?type=nuc&id=151850670916787.9)) | AntH [Streptomyces blastmyceticus] (AGG37770) | 89/94 | epoxidase component |
| *smlI* | [96](http://biosyn.nih.go.jp/2ndfind/show.cgi?type=ami&id=151850670916787.10) ([291](http://biosyn.nih.go.jp/2ndfind/show.cgi?type=nuc&id=151850670916787.10)) | AntI [Streptomyces blastmyceticus] (AGG37771) | 89/93 | epoxidase component |
| *smlJ* | [249](http://biosyn.nih.go.jp/2ndfind/show.cgi?type=ami&id=151850670916787.11)([750](http://biosyn.nih.go.jp/2ndfind/show.cgi?type=nuc&id=151850670916787.11)) | Ant2J [Streptomyces blastmyceticus] (BAM21055) | 75/80 | epoxidase component |
| *smlK* | [165](http://biosyn.nih.go.jp/2ndfind/show.cgi?type=ami&id=151850670916787.12) ([498](http://biosyn.nih.go.jp/2ndfind/show.cgi?type=nuc&id=151850670916787.12)) | AntK [Streptomyces blastmyceticus] (AGG37773) | 81/89 | epoxidase component |
| *smlL* | [364](http://biosyn.nih.go.jp/2ndfind/show.cgi?type=ami&id=151850670916787.13) ([1095](http://biosyn.nih.go.jp/2ndfind/show.cgi?type=nuc&id=151850670916787.13)) | AntL [Streptomyces blastmyceticus] (AGG37774) | 76/81 | epoxidase component |
| *smlM* | [269](http://biosyn.nih.go.jp/2ndfind/show.cgi?type=ami&id=151850670916787.14) ([810](http://biosyn.nih.go.jp/2ndfind/show.cgi?type=nuc&id=151850670916787.14)) | AntN [Streptomyces blastmyceticus] (AGG37776) | 78/86 | tryptophan dioxygenase |
| *smlN* | [270](http://biosyn.nih.go.jp/2ndfind/show.cgi?type=ami&id=151850670916787.15)([813](http://biosyn.nih.go.jp/2ndfind/show.cgi?type=nuc&id=151850670916787.15)) | AntQ [Streptomyces blastmyceticus] (AGG37777) | 79/86 | kynurenine formamidase |
| *smlO* | [418](http://biosyn.nih.go.jp/2ndfind/show.cgi?type=ami&id=151850670916787.16) ([1257](http://biosyn.nih.go.jp/2ndfind/show.cgi?type=nuc&id=151850670916787.16)) | Ant2O [Streptomyces blastmyceticus] (BAM21061) | 80/88 | kynureninase |
| *smlP* | [258](http://biosyn.nih.go.jp/2ndfind/show.cgi?type=ami&id=151850670916787.17) ([777](http://biosyn.nih.go.jp/2ndfind/show.cgi?type=nuc&id=151850670916787.17)) | aminotransferase [Streptomyces sp. SCSIO] (WP_086162208) | 66/78 | Aminotranferase |
| *orf-2* | [153](http://biosyn.nih.go.jp/2ndfind/show.cgi?type=ami&id=151850670916787.18) ([463](http://biosyn.nih.go.jp/2ndfind/show.cgi?type=nuc&id=151850670916787.18)) | Chitinase [Streptomyces albireticuli]  (ARZ71893) | 97/97 | chitinase |

**Supplementary Figure 1.** Gene organization of *sml* cluster

| Gene | Amino acid  (base pairs) | Protein homologue  [strain] (Accession number) | Identity /Similarity (%) | Proposed function |
| --- | --- | --- | --- | --- |
| *orf-1* | [455](http://biosyn.nih.go.jp/2ndfind/show.cgi?type=ami&id=151850785519529.1) ([1368](http://biosyn.nih.go.jp/2ndfind/show.cgi?type=nuc&id=151850785519529.1)) | Hypothetical protein [Streptomyces sp. TLI_146] ([PKV90154](https://www.ncbi.nlm.nih.gov/nuccore/61212710)) | 36/50 | hypothetical protein |
| *natA* | [175](http://biosyn.nih.go.jp/2ndfind/show.cgi?type=ami&id=151850785519529.2) ([528](http://biosyn.nih.go.jp/2ndfind/show.cgi?type=nuc&id=151850785519529.2)) | Ant2A [Streptomyces blastmyceticus] ([BAM21045](https://www.ncbi.nlm.nih.gov/nuccore/1173163)) | 83/91 | sigma factor |
| *natB* | [4619](http://biosyn.nih.go.jp/2ndfind/show.cgi?type=ami&id=151850785519529.3) ([13860](http://biosyn.nih.go.jp/2ndfind/show.cgi?type=nuc&id=151850785519529.3)) | AntC [Streptomyces blastmyceticus] (AGG37764) | 57/66 | NRPS (C-A-T,C-A-KR-T,C-A-KR-T) |
| *natC* | [1411](http://biosyn.nih.go.jp/2ndfind/show.cgi?type=ami&id=151850785519529.4) ([4236](http://biosyn.nih.go.jp/2ndfind/show.cgi?type=nuc&id=151850785519529.4)) | Hypothetical protein ACZ90_45135 [Streptomyces albus subsp. albus] (KUJ65684) | 75/82 | PKS  (KS-AT-MT-T-TE) |
| *natD* | 2403 (7212) | KR-domain containing protein [Streptomyces] (WP_051829854) | 44/55 | NRPS  (C-A-KR-T-TE) |
| *natE* | [65](http://biosyn.nih.go.jp/2ndfind/show.cgi?type=ami&id=151850785519529.7) ([198](http://biosyn.nih.go.jp/2ndfind/show.cgi?type=nuc&id=151850785519529.7)) | MbtH [ Actinobacteria bacterium OK074] ( WP_054216417) | 67/78 | MbtH |
| *natF* | [342](http://biosyn.nih.go.jp/2ndfind/show.cgi?type=ami&id=151850785519529.8) ([1029](http://biosyn.nih.go.jp/2ndfind/show.cgi?type=nuc&id=151850785519529.8)) | NAD-dependent epimerase [Nonomurae coxensis] ([WP_033408890](https://www.ncbi.nlm.nih.gov/nuccore/75313128)) | 54/65 | NAD-dependent reductase |
| *natG* | [255](http://biosyn.nih.go.jp/2ndfind/show.cgi?type=ami&id=151850785519529.9) ([768](http://biosyn.nih.go.jp/2ndfind/show.cgi?type=nuc&id=151850785519529.9)) | thioesterase [Streptomyces caatingaensis] ([WP_049714988](https://www.ncbi.nlm.nih.gov/nuccore/121652)) | 69/78 | Thioesterase |
| *natH* | [231](http://biosyn.nih.go.jp/2ndfind/show.cgi?type=ami&id=151850785519529.10) ([696](http://biosyn.nih.go.jp/2ndfind/show.cgi?type=nuc&id=151850785519529.10)) | 4'-phosphopantetheinyl transferase EntD [Streptomyces sp. TLI_146]  ( PKV83804) | 59/66 | 4'-phosphopantetheinyl transferase |
| *natI* | [495](http://biosyn.nih.go.jp/2ndfind/show.cgi?type=ami&id=151850785519529.11) ([1488](http://biosyn.nih.go.jp/2ndfind/show.cgi?type=nuc&id=151850785519529.11)) | Ant2F [Streptomyces blastmyceticus] ([BAM21051](https://www.ncbi.nlm.nih.gov/nuccore/1173163)) | 80/87 | AMP-ligase |
| *natJ* | [73](http://biosyn.nih.go.jp/2ndfind/show.cgi?type=ami&id=151850785519529.12) ([222](http://biosyn.nih.go.jp/2ndfind/show.cgi?type=nuc&id=151850785519529.12)) | Ant2G [Streptomyces blastmyceticus] (BAM21052) | 96/95 | Carrier protein |
| *natK* | [333](http://biosyn.nih.go.jp/2ndfind/show.cgi?type=ami&id=151850785519529.13) ([1002](http://biosyn.nih.go.jp/2ndfind/show.cgi?type=nuc&id=151850785519529.13)) | AntH [Streptomyces blastmyceticus] (AGG37770) | 90/95 | epoxidase component |
| *natL* | [97](http://biosyn.nih.go.jp/2ndfind/show.cgi?type=ami&id=151850785519529.14) ([294](http://biosyn.nih.go.jp/2ndfind/show.cgi?type=nuc&id=151850785519529.14)) | AntI [Streptomyces blastmyceticus] (AGG37771) | 82/91 | epoxidase component |
| *natM* | [251](http://biosyn.nih.go.jp/2ndfind/show.cgi?type=ami&id=151850785519529.15) ([756](http://biosyn.nih.go.jp/2ndfind/show.cgi?type=nuc&id=151850785519529.15)) | Ant2J [Streptomyces blastmyceticus] (BAM21055) | 71/79 | epoxidase component |
| *natN* | [165](http://biosyn.nih.go.jp/2ndfind/show.cgi?type=ami&id=151850785519529.16) ([498](http://biosyn.nih.go.jp/2ndfind/show.cgi?type=nuc&id=151850785519529.16)) | AntK [Streptomyces blastmyceticus] (AGG37773) | 83/89 | epoxidase component |
| *natO* | [366](http://biosyn.nih.go.jp/2ndfind/show.cgi?type=ami&id=151850785519529.17) ([1101](http://biosyn.nih.go.jp/2ndfind/show.cgi?type=nuc&id=151850785519529.17)) | AntL [Streptomyces blastmyceticus] (AGG37774) | 74/82 | epoxidase component |
| *natP* | [270](http://biosyn.nih.go.jp/2ndfind/show.cgi?type=ami&id=151850785519529.18) ([813](http://biosyn.nih.go.jp/2ndfind/show.cgi?type=nuc&id=151850785519529.18)) | AntN [Streptomyces blastmyceticus] (AGG37776) | 79/87 | tryptophan dioxygenase |
| *natQ* | [274](http://biosyn.nih.go.jp/2ndfind/show.cgi?type=ami&id=151850785519529.19) ([825](http://biosyn.nih.go.jp/2ndfind/show.cgi?type=nuc&id=151850785519529.19)) | AntQ [Streptomyces blastmyceticus] (BAM21060) | 76/83 | kynurenine formamidase |
| *natR* | [416](http://biosyn.nih.go.jp/2ndfind/show.cgi?type=ami&id=151850785519529.20) ([1251](http://biosyn.nih.go.jp/2ndfind/show.cgi?type=nuc&id=151850785519529.20)) | Ant2O [Streptomyces blastmyceticus] (BAM21061) | 80/87 | kynureninase |
| *orf-2* | [588](http://biosyn.nih.go.jp/2ndfind/show.cgi?type=ami&id=151850785519529.21) ([1767](http://biosyn.nih.go.jp/2ndfind/show.cgi?type=nuc&id=151850785519529.21)) | Phospholipase [Streptomyces sp. NRRL F-4489] (WP_066981186) | 70/79 | Phosphoesterase |

**Supplementary Figure 2.** Gene organization of *nat* cluster

Comparison with the previously published annotation reveals that NatD and NatE varies between the two studies - the identically named genes are located in opposite reading frames and have dissimilar functions (MbtH in this study and PKS in the previous). The direction and size of NatG-NatR are also different in each study.

**Supplementary Figure 3.** HPLC analyses of the metabolites from *S. lividans* TK21 i) and transformants harboring ii) pKU518J06 and ii) pKU518nant. The chromatogram represents the UV absorbance at 320 nm. * and ** indicate unidentified analogs with *m/z* values that were 14 and 28 less than **3b**, respectively.


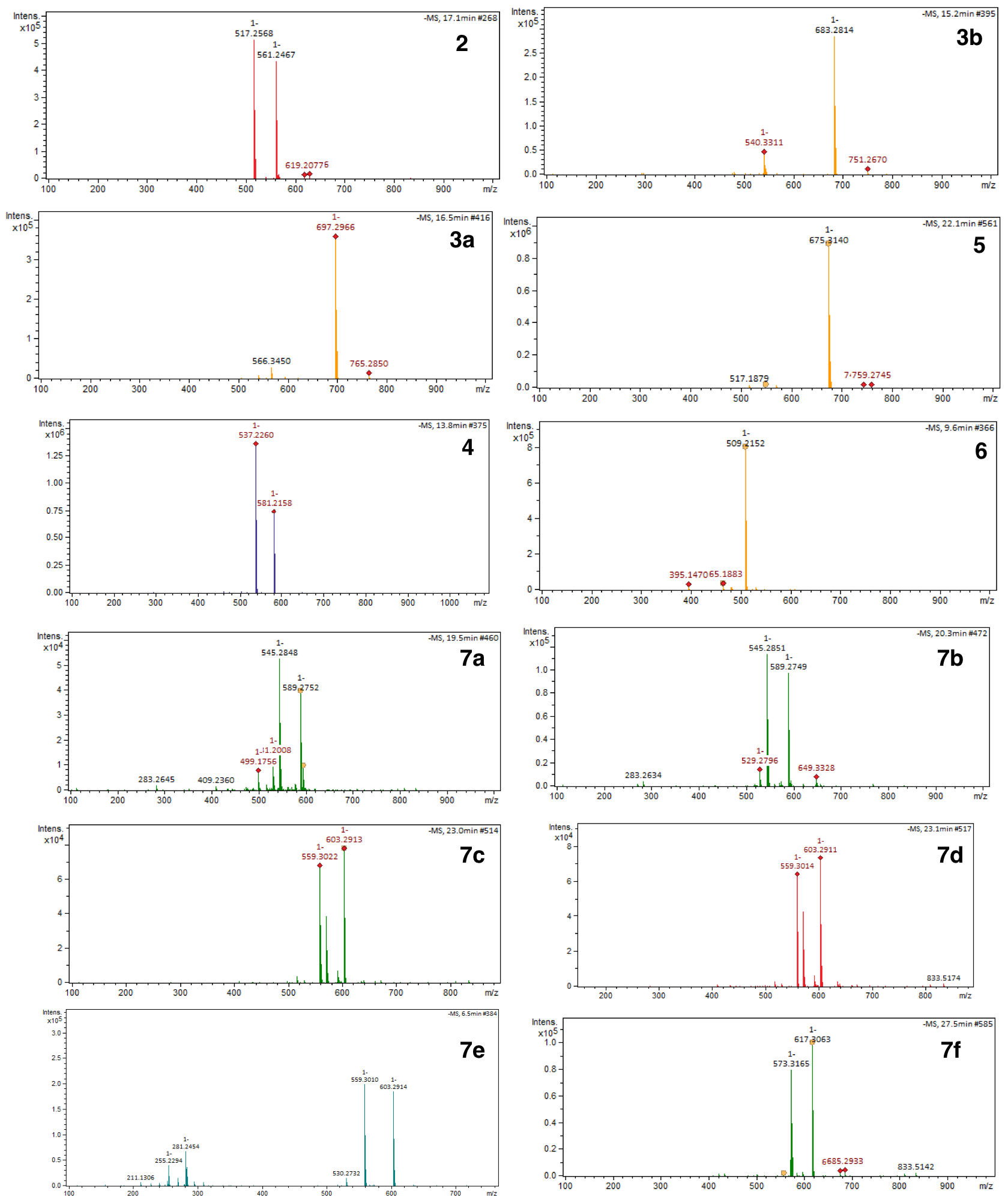


**Supplementary Figure 4.** MS spectra of the isolated compounds

**Supplementary Figure 5. ^1^H NMR spectrum of JBIR-06 (2)**

**Supplementary Figure 6. ^1^H NMR spectrum of neoantimycin A (3a)**

**Supplementary Figure 7. ^1^H NMR spectrum of neoantimycin F (3b)**


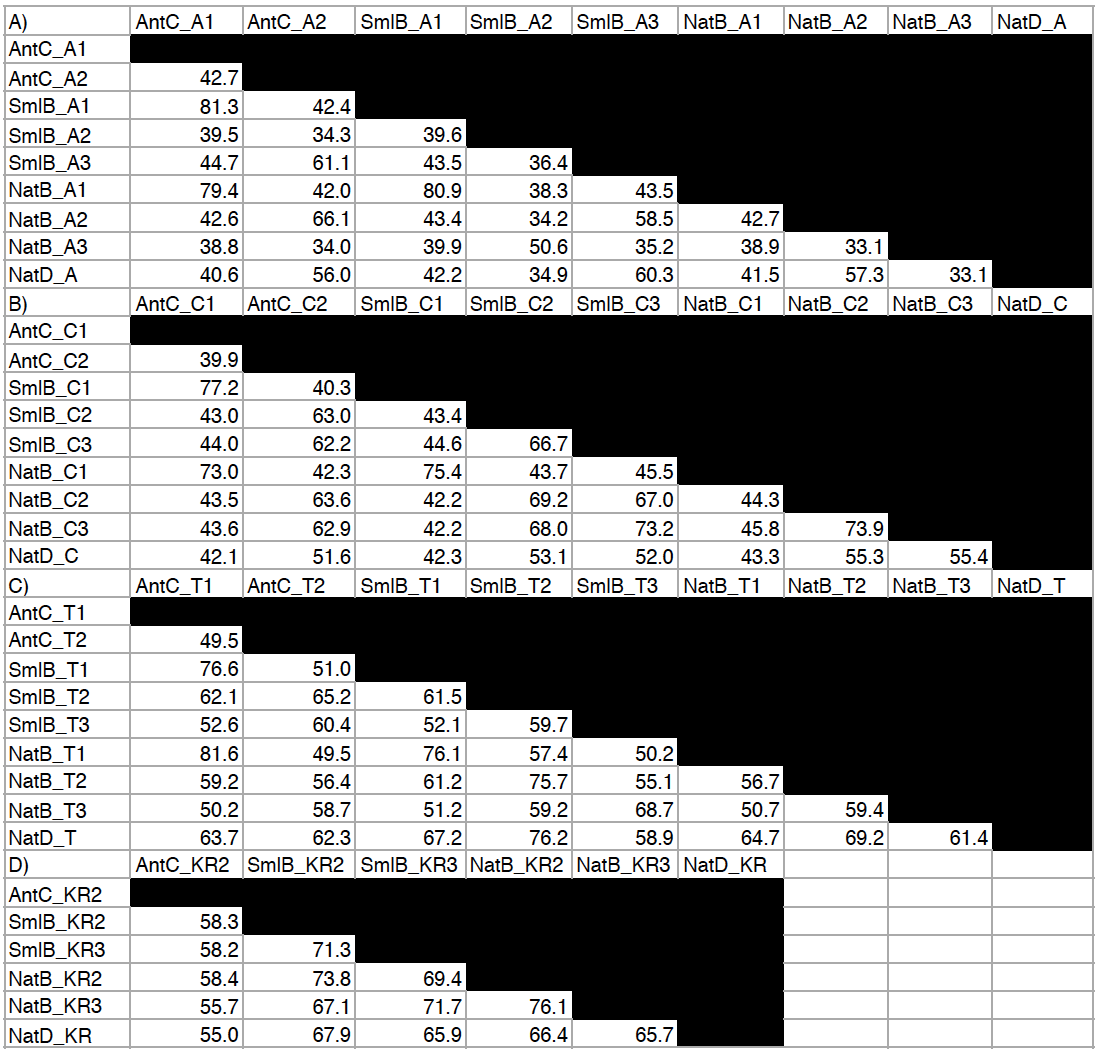


**Supplementary Figure 8. continued**

**Supplementary Figure 8.** Genetic identity map of the nucleotide alignment of the adenylation (A), condensation (B), thiolation (C), ketoreductase (D) domains of the antimycin (*ant*), JBIR-06 (*sml*), and neoantimycin (*nat*) NRPS modules. The alignment was done by muscle alignment with default parameters by Geneious. The numeric numbers indicate the identity (%). The identity values for A (E), C (F), T (G), and KR (H) domain against the closest ancestor homologs are also shown. The identities more than 65% are coloured red. The number after domain abbreviation corresponds to the number of module. (e.g. “A1” means the adenylation domain in the first module.)

**Supplementary Figure 9.** Alignment of AntD, SmlC, and NatC.

The five amino acids in AT which were mutated in this study are shown in green frame.

PRTELERQVAARWQEVLGVEPVGVEDNFFDLGGDSLLALQLVTRLRDELKAELSVKRLLERLTVAAVAQDIAGGGGDSAGALDVVLKLRSGGSGTPLFCVHPAGGVSWPYARLLPAIDERFPVYGIQSRGLTDPGSMPRTIEEMAADYVQEIRAVQPSGPYALVGWSLGGLVAHAMAGQLERAGERVALLATLDAFPFTADDVLKLPDADEVNAFLMQVLLTDSGITAGPDEPAPDLDEVLARLRGSNSVMSGIEAETLARIADVMLTNTRVLFGYTPEAISGDLLAFSAAESHDAAEPAPATRWKPYVGGAVESRDLPCDHYNVLRGEALGIVGQELGE

**Supplementary Figure 10.** The amino acid sequence for the Hhomology modeling of ACP_NatC-_TE_SmlC_

We connect the NatC (1320-1390, colored red) with SmlC (1372-1646, colored blue) as shown below, and submit it to Swiss Model (<https://swissmodel.expasy.org>). The model of ACP_NatC-_TE_SmlC_ domains was constructed, based on the NRPS module from *Acinetobacter baumannii* (PBD: 4ZXH), which is automatically chosen as a template by swissmodel.


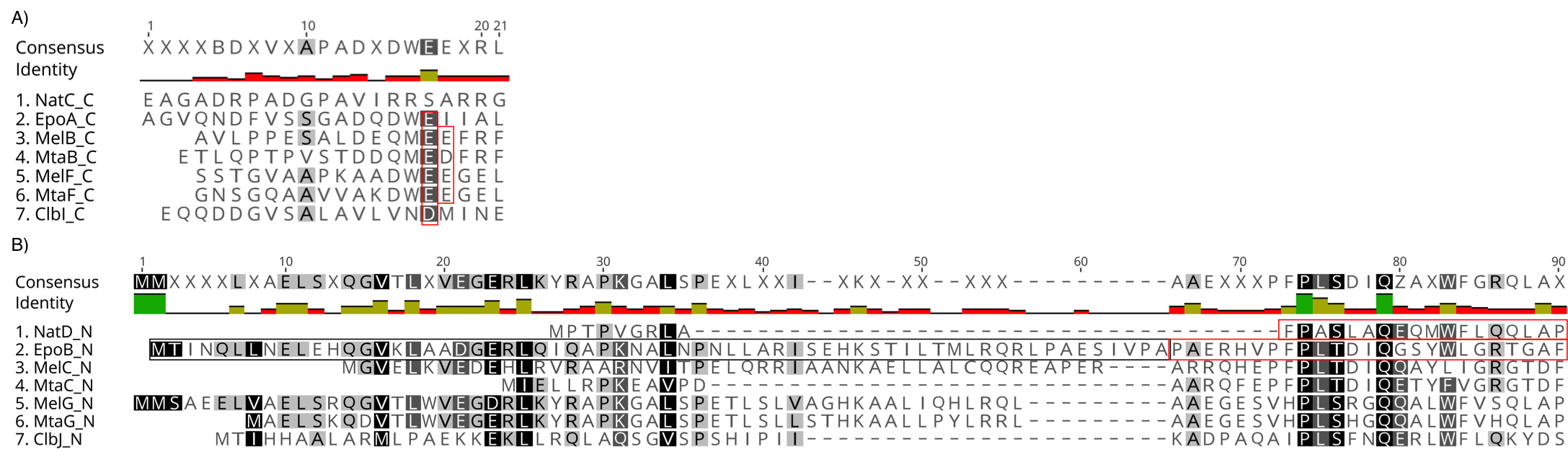


**Supplementary Figure 11.** Alignments of C-terminal of cis-AT PKS modules which interact with NRPS modules encoded in the distinct proteins (A) and N-terminal of NRPS modules which interact with cis-AT PKS modules (B)

The alignment was done by muscle alignment with default parameters by Geneious. (A) The red frame indicates the conserved acidic residues. (B)The black and red frames indicate the N-terminal docking domain and NRPS domain, respectively, shown in Dowling, D. P. *et al.* *Proc. Natl. Acad. Sci.* **113,** 12432–12437 (2016). The red frame in NatD indicates the head of C domain predicted by PKS-NRPS analysis website (<http://nrps.igs.umaryland.edu>). Abbreviation: MtaB (accession number: Q9RFL0) and MtaC (Q9RFK9) (PKS and NRPS in myxothiazol biosynthesis), ClbI (CDO15373) and ClbJ (CDO15372) (PKS and NRPS in colibactin biosynthesis), EpoA (Q9KJ00) and EpoB (Q9KIZ9) (PKS and NRPS in epothilone biosynthesis), and MtaF (Q9RFK6), MtaG (Q9RFK5) (PKS and NRPS in myxothiazol biosynthesis), and MelB (CAD89773), MelC (CAD89774), MelF (CAD89777), and MelG (CAD89778) (PKSs and NRPSs in melithiazol biosynthesis).

.


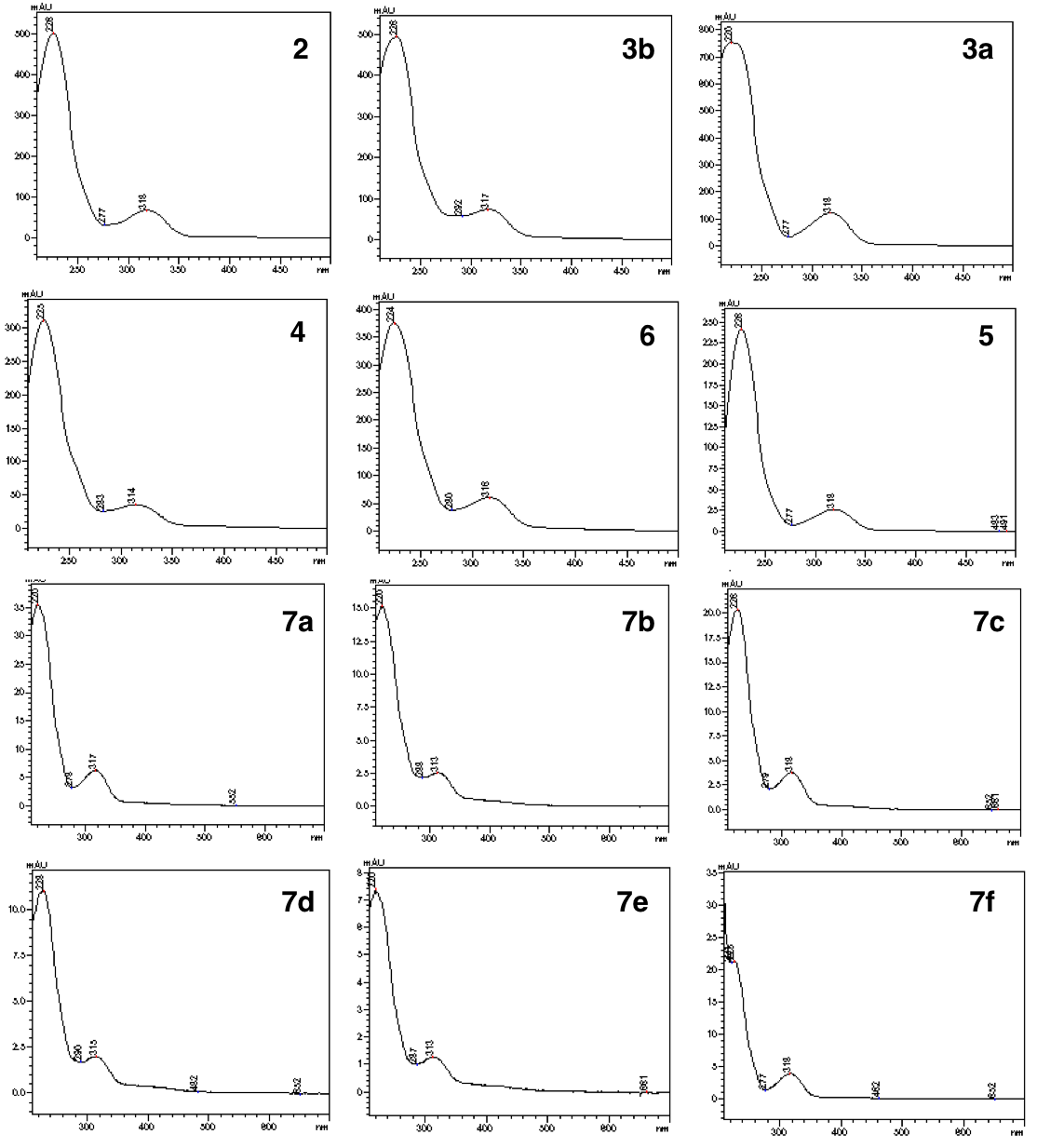


**Supplementary Figure 12.** UV spectra of the isolated compounds

**Supplementary Figure 13. ^1^H NMR spectrum of 4**

**Supplementary Figure 14. ^13^C NMR spectrum of 4**

**Supplementary Figure 15. COSY spectrum of 4**

**Supplementary Figure 16. HMQC spectrum of 4**

**Supplementary Figure 17. HMBC spectrum of 4**

**Supplementary Figure 18.** Key COSY and HMBC correlations for structure elucidation

**Supplementary Figure 19. The design of in-fusion reaction to construct pZH2-NatD**

**Supplementary Figure 20. ^1^H NMR spectrum of 5**

**Supplementary Figure 21. ^13^C NMR spectrum of 5**

**Supplementary Figure 22. COSY spectrum of 5**

**Supplementary Figure 23. HMQC spectrum of 5**

**Supplementary Figure 24. HMBC spectrum of 5**

**Supplementary Figure 25. ^1^H NMR spectrum of 6**

**Supplementary Figure 26. ^13^C NMR spectrum of 6**

**Supplementary Figure 27. COSY spectrum of 6**

**Supplementary Figure 28. HMQC spectrum of 6**

**Supplementary Figure 29. HMBC spectrum of 6**
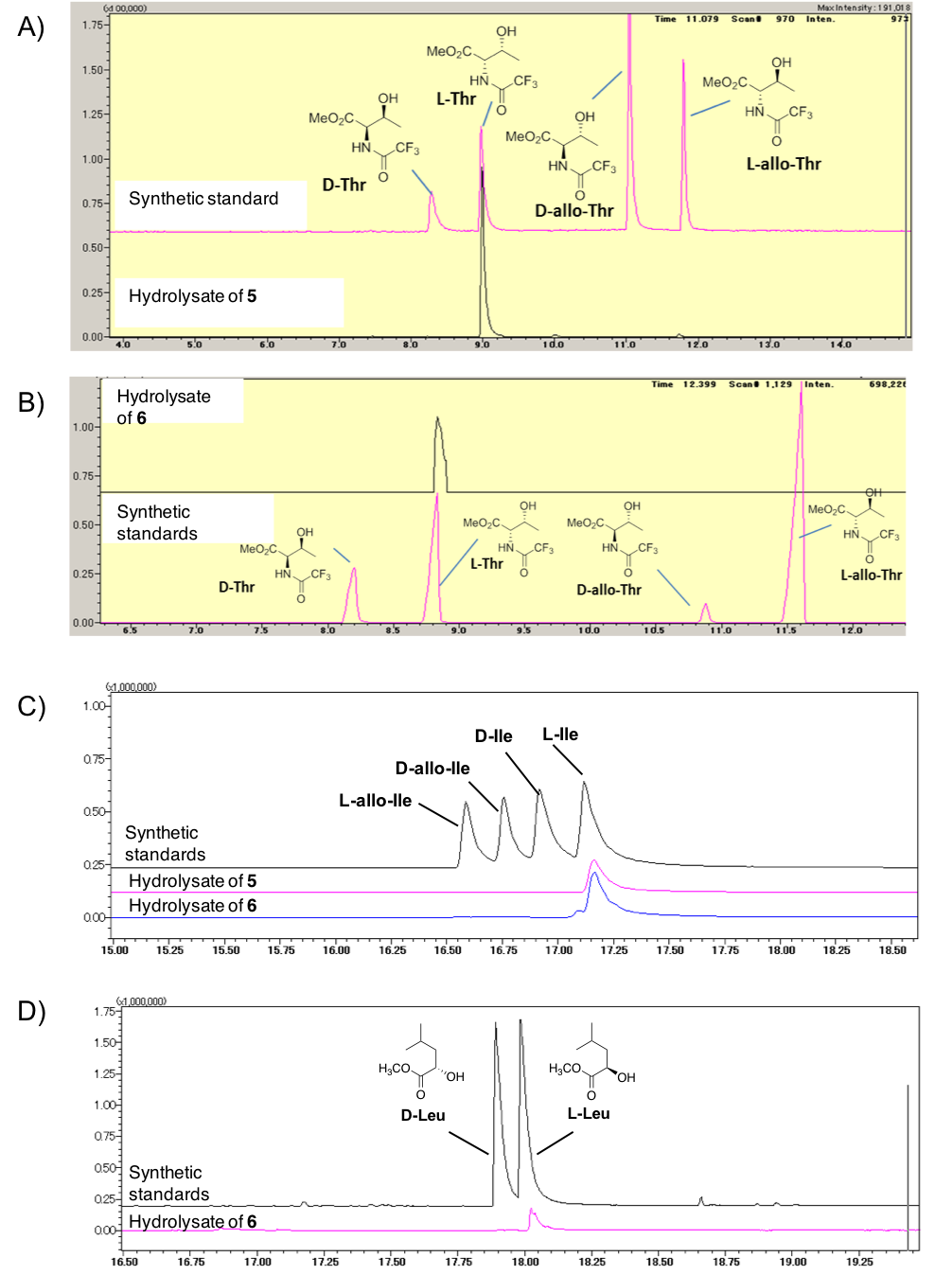


**Supplementary Figure 30.** The identification of stereochemistry of each building block in **5** and **6**

Extracted ion chromatgram m/z 152 (A, B), m/z 90 (C), and m/z 69 (D).

**Supplementary Figure 31. ^1^H NMR spectrum of 7a**

**Supplementary Figure 32. ^13^C NMR spectrum of 7a**

**Supplementary Figure 33. COSY spectrum of 7a**

**Supplementary Figure 34. HMQC spectrum of 7a**

**Supplementary Figure 35. HMBC spectrum of 7a**

**Supplementary Figure 36. ^1^H NMR spectrum of 7c**

**Supplementary Figure 37. ^13^C NMR spectrum of 7c**

**Supplementary Figure 38. COSY spectrum of 7c**

**Supplementary Figure 39. HMQC spectrum of 7c**

**Supplementary Figure 40. HMBC spectrum of 7c**

**Supplementary Figure 41. ^1^H NMR spectrum of 7f**

**Supplementary Figure 42. ^13^C NMR spectrum of 7f**

**Supplementary Figure 43. COSY spectrum of 7f**

**Supplementary Figure 44. HMQC spectrum of 7f**

**Supplementary Figure 45. HMBC spectrum of 7f**

**Supplementary Figure 46. ^1^H NMR spectrum of 7b**

**Supplementary Figure 47. COSY spectrum of 7b**

**Supplementary Figure 48. ^1^H NMR spectrum of 7d**

**Supplementary Figure 49. COSY spectrum of 7d**

**Supplementary Figure 50. ^1^H NMR spectrum of 7e**

**Supplementary Figure 51. COSY spectrum of 7e**

**
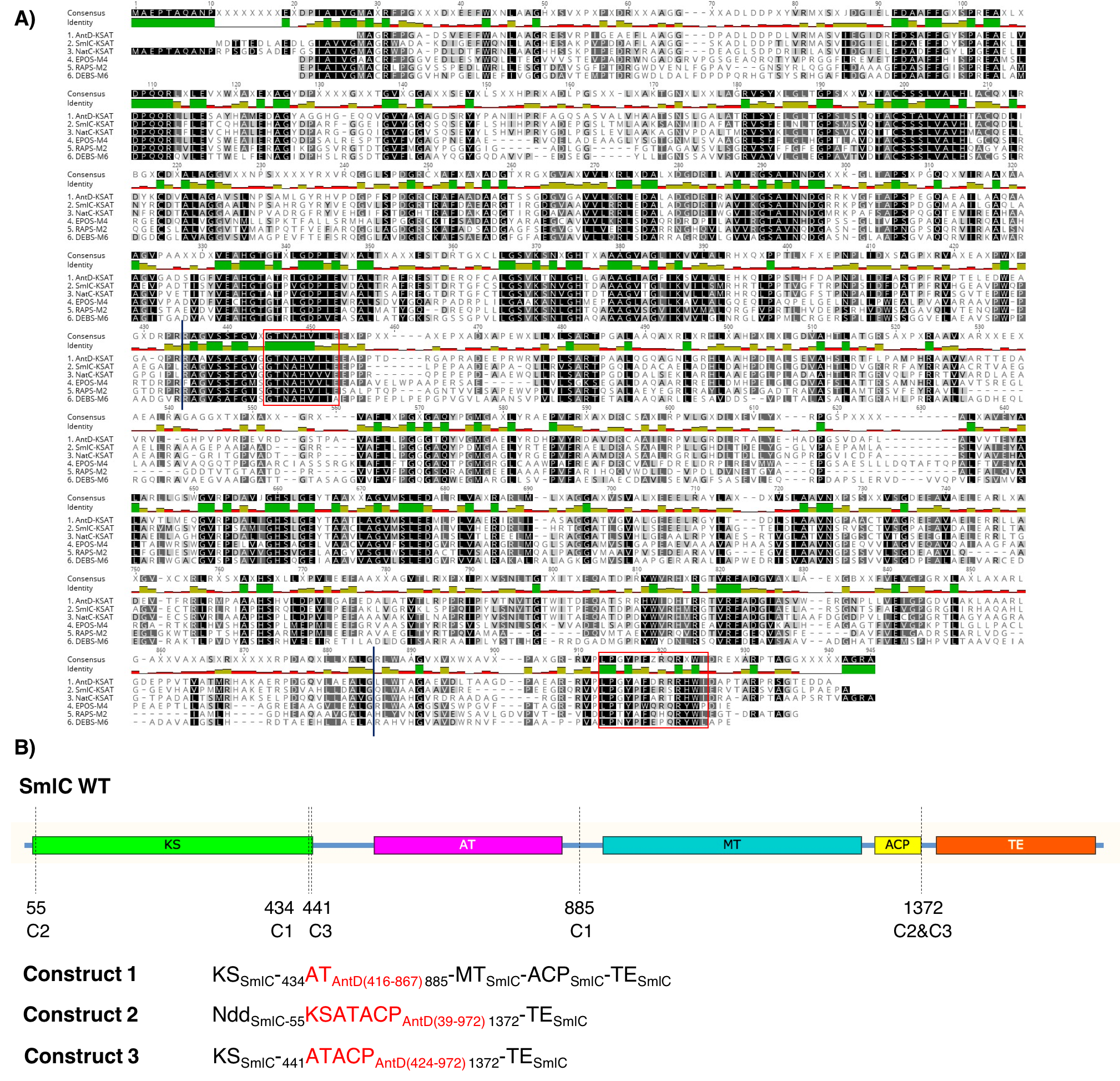
**

**Supplementary Figure 52.** The domain swapping carried out in this study

1. Alignment of N-terminal region (including KS and AT domains) of AntD, SmlC, NatC, and the related PKS modules. Red frame indicates the ‘optimal’ fusion junctions identified in Yuzawa, S. *et al.* *ACS Synth. Biol.* **6,** 139–147 (2017). Blue line indicates the fusion point used in the construct 1 shown in B) The alignment was done by muscle alignment with default parameters by Geneious.
2. The SmlC-AntD fused construct by domain swapping. Black indicates the domain and amino acid number of SmlC, and Red indicates those of AntD.

**Supplementary Table 1.** The plasmids constructed in this study

| Plasmids | purpose |
| --- | --- |
| pKU518 | A BAC vector constructed by Dr. H. Ikeda’s group |
| pUC19 | A common cloning vector |
| pIJ773 | A vector harboring AprR resistance gene |
| pTYM19ep | A vector which harboring ermE promoter and ΦC31 sequence, constructed by Dr. H. Onaka’s group |
| pKU518J06 | A BAC vector harboring JBIR-06 (sml) gene cluster |
| pKU518nant | A BAC vector harboring naoantimycin (nat) gene cluster |
| pUC19-SmlCTE-AprR-Perm | pUC19 harboring *smlC*TE, AprR, and ermE promoter (Perm) |
| pKU518nantΔ*natD::smlCTE* | A pKU518nant engineered by deleting *natD* and introducing a fragment, *smlC*TE-AprR-Perm (from pUC19-SmlCTE-AprR-Perm) in place |
| pUC19-CtNatCAprRPerm | pUC19 harboring C-terminus of *natC* , AprR, and Perm |
| pKU518J06Δ*smlCTE* | A pKU518J06 engineered by deleting *smlCTE* and introducing a fragment C-terminus of *natC* , AprR, and Perm (from pUC19-CtNatCAprRPerm) |
| pZH2-NatD | A φC31 integration vector to express natD, consisted of pUC19, AprR and PermE |
| pUC19-NatDfixed | A vector harboring *natD* engineered to possess multiple nonsense mutation to avoid unwanted recombination between *natB* and *D* |
| pKU518J06**Δ**smlC | A pKU518J06 engineered by deleting *SmlC* and introducing a fragment, AprR, and Perm (from pUC19-CtNatCAprRPerm) |
| pZH2-SmlCAT_ant_-AntEV350G | A φC31 integration vector to express SmlC including 5AA mutation which tolerates substrate specificity, and AntEV350G |

**Supplementary Table 2.** The primers used in this study

Underline for in-fusion reaction, Italic for Red/ET reaction, the blue sequences were used to integrated with pUC19 cut by EcoRI-HindIII for subcloning, and the red sequences were used for recombination each other through in-fusion.

| Primer | sequence |
| --- | --- |
| I | tgattacgccaagctGCCGGGGGCGGCGGGGACTCG |
| II | CGCTCGAACTCACTGTCAGGCCAGCCGGTTCAGGCGCTC |
| III | tgattacgccaagcttgaataggaacttatgagctcagc |
| IV | cgagcgaaatgaccggcccacagaatgatgtcacgctg |
| V | cataagttcctattcGCTGGCGAAAGGGGGATGTGC |
| VI | gacggccagtgaattcATGTGGATCCTACCAACCGGCAC |
| VII | *tcaagcgcctgctggagcggctgaccgtggccgccgtggcccaggacatc*GCCGGGGGCGGCGGGGACTCG |
| VIII | *CAGAGCGCGGAAGCTCAGGTTCTCGTTGTCGAAGGGACTGGTGGATGTCA*ATGTGGATCCTACCAACCGGCAC |
| IX | gacggccagtgaattcgaggccggcgccgaccgcccggccgac |
| X | ggtgataaacttatcCATgcgagggactcctggacgg |
| XI | gataagtttatcaccaccgac |
| XII | gaataggaacttatgagctcagccaatc |
| XIII | cataagttcctattcTGAATTGCAGGTCCAGGAAG |
| XIV | tgagagtgcaccatatgGGTCCTCCTGTGGAGTGGTTCTGTGGATCCTACCAACCGGCAC |
| XV | *TGGCCGCGCTCGTGGAGCACCCCACGGTGGCGGGCCTGGCGGCGCACCTC*gaggccggcgccgaccgcccggccgac |
| XVI | *TGGCCGACGATGCCGAGCGCCTCGCCGCGCAGCACGTTGTAGTGGTCG*catatgGGTCCTCCTGTGGAGTGG |
| XVII | CACAGGAGGACCcatATGCCAACCCCCGTAGGCCGTC |
| XVIII | tgagagtgcaccatatgacgaagggactggtggatgTCACG |
| XIX | *GGCAATGGACACCACTGAGGACCTCGCCGAGGACCTCGGCATCGCCGTGG*gcccacagaatgatgtcacgctg |
| XX | *ACACCCTGCGTGGCCGGGTTGAGCGTGGCCCTCGCCTGATGGTTATT*CATatgGGTCCTCCTGTGGAGTGGTTCTG |
| XXI | CACAGGAGGACCcatATGGACACCACTGAGGACCTCGCCGAGG |
| XXII | cgcggcggccggcatCCGGATCCTGGTGCACTCGACC |
| XXIII | atgccggccgccgcgCACTCGCGGCAACTGGACGAGG |
| XXIV | tgagagtgcaccataCCCGGGTGAACGGTCAGGCCAG |
| XXV | tgattacgccaagcttGTTGTAAAACGACGGCCAGTG |
| XXVI | GTCAGTGATGAAGCTTGGAAACAGCTATGACCATGATTACG |
| 1f | GACGGCCAGTGAATTCGAGGCCGGCGCCGACCGCCCGGCCGAC |
| 1r | ATCAAATAGATCCGTACTATAGACGACCAGCACGCCCAG |
| 2f | CGGATCTATTTGATGCGCCGACGGTACGCCGGCTG |
| 2r | TGATTACGCCAAGCTTCCTCGTCCAGCACCGGCAGC/CATGCCGCCGCGAGTTCCTCGTCCAGCACCGGCAGC |
| 3f | ACTCGCGGCGGCATGGGAGCGGCACCTGGTGGAGAG |
| 3r | TGATCCAAAGGCATCCAATTAAAGGACCGGTCGGCCG |
| 4f | ATGCCTTTGGATCATGTCGGTGGC |
| 4r | CAAGGCGAGTCGCCAAGAGAGAC |
| 5f | GACGGCCAGTGAATTCGTCAGCCGGATGCGCACCGCTC/ TGGCGACTCGCCTTGTCAGCCGGATGCGCACCGCTC |
| 5r | CATCATTCTGTGGGCTCATGCCACGGCCTCCGGGGC |
| Apr-f | GCCCACAGAATGATGTCACGCTG |
| PermE-R | TGATTACGCCAAGCTTATGTGGATCCTACCAACCGGCACG |
| 3f1 | GACGGCCAGTGAATTCTGGGAGCGGCACCTGGTGGAGAG |
| 3r1 | GCATCTTCTGCTAGTCGGGCATAGCTCTGCCGGTCC |
| 3f2 | CGACTAGCAGAAGATGCCTCCCGGGTGCTGGGCG |
| 3r2 | TGATTACGCCAAGCTTGACACATAGTCCCGGGTGCCG |
| 3f3 | GACGGCCAGTGAATTCGTCATAGGAGGTTTCGCCGCCGTCCCGCTC |
| 3r3 | TCTATTGCTGCTACTCGGGGGGCGGGCC |
| 3f4 | AGTAGCAGCAATAGATGAACTGCTGTCCGGGGATCCG |
| 3r4 | GACTGCTTTGGGGCGACCCGTACTTCCCGACGTGAGCAGCAGCAGGACG |
| 3f5 | CGCCCCAAAGCAGTCGAACAACGTCATCGTAATATCCTCACCCAGG |
| 3r5 | TGATTACGCCAAGCTTAGGACCGGTCGGCCGGGGTGATC |

**Supplementary Table 3.** The HR-MS data of isolated compounds

|  | **HR-MS** |
| --- | --- |
| **2** | 561.2467 (Cal. 561.2454, C_28_H_37_N_2_O_10_^-^) |
| **3a** | 697.2966 (Cal. 697.2978, C_36_H_45_N_2_O_12_^-^) |
| **3b** | 683.2814 (Cal. 683.2821, C_35_H_43_N_2_O_12_^-^) |
| **4** | 581.2158 (Cal. 581.2141, C_30_H_33_N_2_O_10_^-^) |
| **2** | 561.2467 (Cal. 561.2454, C_28_H_37_N_2_O_10_^-^) |
| **5** | 675.3140 (Cal. 675.3134, C_34_H_47_N_2_O_12_^-^) |
| **6** | 509.2152 (Cal. 509.2141, C_24_H_33_N_2_O_10_^-^) |
| **7a** | 589.2752 (Cal. 589.2767, C_30_H_41_N_2_O_10_^-^) |
| **7b** | 589.2749 (Cal. 589.2767, C_30_H_41_N_2_O_10_^-^) |
| **7c** | 603.2913 (Cal. 603.2923, C_31_H_43_N_2_O_10_^-^) |
| **7d** | 603.2911 (Cal. 603.2923, C_31_H_43_N_2_O_10_^-^) |
| **7e** | 603.2914 (Cal. 603.2923, C_31_H_43_N_2_O_10_^-^) |
| **7f** | 617.3063 (Cal. 617.3080, C_32_H_45_N_2_O_10_^-^) |

**Supplementary Table 4.** ^1^H and ^13^C NMR data of **4**

| Position | ^1^H (multiplet, J value [Hz]) | ^13^C |
| --- | --- | --- |
| 1 | - | 203.4 |
| 2 | 5.70 (t, 5.5) | 79.2 |
| 3 | - | 165.6 |
| 4 | 5.19 (d, 7.5) | 77.6 |
| 5 | - | 168.4 |
| 6 | 5.23 (dd, 8.5, 3.0) | 55.4 |
| 7 | 5.85 (qd, 6.5, 3.0) | 71.0 |
| 8 | - | 171.9 |
| 9 | - | 53.6 |
| 10a | 3.29 (dd, 14.0, 5.5) | 37.8 |
| 10b | 3.12 (dd, 14.0, 6.0) | 37.8 |
| 11 | - | 135.5 |
| 12 | 7.12 (d, 7.5) | 130.0 |
| 13 | 7.27 (m) | 128.6 |
| 14 | 7.22 (d, 7.0) | 127.3 |
| 15 | 7.27 (m) | 128.6 |
| 16 | 7.12 (d, 7.5) | 130.0 |
| 17 | 2.29 (m) | 28.8 |
| 18 | 0.94 (d, 7.0) | 18.5 |
| 19 | 0.86 (d, 7.0) | 17.2 |
| 20 | - | 170.4 |
| 21 | - | 112.8 |
| 22 | - | 150.6 |
| 23 | - | 127.5 |
| 24 | 8.58 (d, 8.0) | 125.0 |
| 25 | 6.96 (m) | 119.2 |
| 26 | 7.29 (m) | 120.3 |
| 27 | 8.51 (s) | 159.0 |
| 28 | 1.27 (d, 6.5) | 16.2 |
| 29 | 1.29 (s) | 23.0 |
| 30 | 1.09 (s) | 20.3 |
| 6-NH | 6.95 (m) | - |
| 22-OH | 12.53 (s) | - |
| 23-NH | 7.93 (s) | - |

| Position | ^1^H (multiplet, J value [Hz]) | ^13^C |
| --- | --- | --- |
| 1 | - | 203.3 |
| 2 | 5.33 (dd, 10.5, 1.5) | 74.7 |
| 3 | - | 168.4 |
| 4 | 5.04 (d, 3.0) | 77.4 |
| 5 | - | 168.8 |
| 6 | 5.13 (dd, 9.0, 2.5) | 55.3 |
| 7 | 5.74 (qd, 6.5, 2.5) | 71.4 |
| 8 | - | 168.0 |
| 9 | 5.24 (dd, 8.0, 3.0) | 76.5 |
| 10 | - | 172.0 |
| 11 | - | 56.0 |
| 12 | 1.80. 1.40 (m) | 40.4 |
| 13 | 1.39 (m, 1H) | 24.9 |
| 14 | 0.97 (d, 7.0) | 21.3 |
| 15 | 0.96 (d, 7.0) | 23.5 |
| 16 | 2.08 (m) | 36.7 |
| 17 | 1.30, 1.40 (m) | 24.1 |
| 18 | 0.97 (t, 7.5) | 12.0 |
| 19 | 1.01 (d, 7.0) | 15.4 |
| 20 | - | 170.5 |
| 21 | - | 113.0 |
| 22 | - | 150.7 |
| 23 | - | 127.5 |
| 24 | 8.57 (d, 8.0) | 124.9 |
| 25 | 6.97 (dt, 8.0, 3.0) | 119.2 |
| 26 | 7.37 (d, 8.0) | 120.5 |
| 27 | 8.51 (d, 1.5) | 159.1 |
| 28 | 1.32 (d, 6.5) | 17.1 |
| 29 | 1.43 (s) | 22.9 |
| 30 | 1.41 (s) | 20.7 |
| 31 | 1.92 (m) | 37.5 |
| 32 | 1.45（m） | 25.0 |
| 33 | 0.92 （t, 6.5） | 11.1 |
| 34 | 0.90 (d, 7.0) | 14.2 |
| 6-NH | 7.06 (brd, 9.0) | - |
| 20-OH | 12.5 (brs) | - |
| 21-NH | 7.92 (brs) | - |

**Supplementary Table 5.** ^1^H and ^13^C NMR data of **5**

**Supplementary Table 6.** ^1^H and ^13^C NMR data of **6**

| Position | ^1^H (multiplet, J value [Hz]) | ^13^C |
| --- | --- | --- |
| 1 | - | 174.0 |
| 2 | 5.14 (d, 6.5) | 72.1 |
| 3 | - | 170.0 |
| 4 | 5.09 (d, 2.5) | 77.5 |
| 5 | - | 170.8 |
| 6 | 4.91 (brd, 8.5) | 57.4 |
| 7 | 4.67 (brd, 5.0) | 68.1 |
| 10 | 1.83, 1.72(m) | 39.7 |
| 11 | 1.62 (m) | 24.4 |
| 12 | 0.93 (brs) | 21.5 |
| 13 | 0.96 (brs) | 23.1 |
| 14 | 2.10 (m) | 36.5 |
| 15 | 1.77, 1.36 (m) | 24.8 |
| 16 | 0.95 (m) | 11.6 |
| 17 | 1.06 (d, 6.5) | 15.3 |
| 18 | - | 170.5 |
| 19 | - | 113.1 |
| 20 | - | 150.6 |
| 21 | - | 127.1 |
| 22 | 8.48 (brs) | 124.7 |
| 23 | 6.86 (t, 7.0) | 118.9 |
| 24 | 7.32(brs) | 120.8 |
| 25 | 8.48 (brs) | 159.3 |
| 26 | 1.29 (d, 6.0) | 19.4 |
| 6-NH | 7.25 (brs) | - |
| 21-NH | 7.96 (brs) | - |

| Position | ^1^H (multiplet, J value [Hz]) | ^13^C |
| --- | --- | --- |
| 1 | - | 201.7 |
| 2 | 5.42 (dd, 7.5, 5.0) | 79.4 |
| 3 | - | 167.9 |
| 4 | 5.27 (d, 6.0) | 77.9 |
| 5 | - | 169.0 |
| 6 | 5.28 (d, 8.5, 3.5) | 55.5 |
| 7 | 5.60 (qd, 6.5, 3.5) | 73.2 |
| 8 | - | 169.5 |
| 9 | 3.24 (t, 7.5) | 55.2 |
| 10 | 1.95, 1.70 (m) | 42.0 |
| 11 | 1.61 (m) | 24.8 |
| 12 | 0.944 (d, 7.0) | 22.9 |
| 13 | 0.936 (d, 7.0) | 22.0 |
| 14 | 2.04 (m) | 37.1 |
| 15 | 1.56, 1.32 (m) | 24.8 |
| 16 | 0.94 (t, 7.5) | 11.1 |
| 17 | 0.99 (d, 7.0) | 15.1 |
| 18 | - | 170.4 |
| 19 | - | 112.9 |
| 20 | - | 150.7 |
| 21 | - | 127.6 |
| 22 | 8.58 (d, 8.0) | 125.1 |
| 23 | 6.96 (t, 8.0) | 119.3 |
| 24 | 7.32 (d, 8.0) | 120.3 |
| 25 | 8.51 (d, 1.0) | 159.1 |
| 26 | 1.39 (d, 6.5) | 16.1 |
| 27 | 1.92,1.69 (m) | 25.7 |
| 28 | 1.28 (m) | 29.3 |
| 29 | 1.26 (m) | 22.6 |
| 30 | 0.89 (t, 7.0) | 14.0 |
| 6-NH | 7.07 (d, 8.5) | - |
| 20-OH | 12.63 (brs) | - |
| 21-NH | 7.91 (brs) | - |

**Supplementary Table 7.** ^1^H and ^13^C NMR data of **7a**

**Supplementary Table 8.** ^1^H and ^13^C NMR data of **7c**

| Position | ^1^H (multiplet, J value [Hz]) | ^13^C |
| --- | --- | --- |
| 1 | - | 201.6 |
| 2 | 5.42 (dd, 7.0, 5.0) | 79.4 |
| 3 | - | 167.9 |
| 4 | 5.27 (d, 7.0) | 77.9 |
| 5 | - | 169.0 |
| 6 | 5.28 (d, 8.5, 3.5) | 55.5 |
| 7 | 5.60 (qd, 6.5, 3.5) | 73.2 |
| 8 | - | 169.5 |
| 9 | 3.21 (t, 7.0) | 55.2 |
| 10 | 1.83, 1.62 (m) | 42.0 |
| 11 | 1.57 | 24.8 |
| 12 | 0.95 (d, 7.0) | 22.9 |
| 13 | 0.94 (d, 7.0) | 22.0 |
| 14 | 2.03 (m) | 37.1 |
| 15 | 1.56, 1.32 (m) | 24.9 |
| 16 | 0.94 (m) | 11.1 |
| 17 | 0.99 (d, 7.0) | 15.1 |
| 18 | - | 170.4 |
| 19 | - | 112.9 |
| 20 | - | 150.7 |
| 21 | - | 127.6 |
| 22 | 8.58 (dd, 8.0, 1.0) | 125.1 |
| 23 | 6.96 (dt, 8.0, 2.0) | 119.3 |
| 24 | 7.32 (dd, 8.0, 1.0) | 120.3 |
| 25 | 8.51 (d, 2.0) | 159.1 |
| 26 | 1.39 (d, 7.0) | 16.1 |
| 27 | 1.94,1.68 (m) | 25.7 |
| 28 | 1.17 (m) | 36.0 |
| 29 | 1.57 (m) | 28.0 |
| 30 | 0.88 (d, 7.5) | 22.6 |
| 31 | 0.87 (d, 7.5) | 22.8 |
| 6-NH | 7.07 (d, 8.5) | - |
| 20-OH | 12.4 (brs) | - |
| 21-NH | 7.91 (brs) | - |

**Supplementary Table 9.** ^1^H and ^13^C NMR data of **7f**

| Position | ^1^H (multiplet, J value [Hz]) | ^13^C |
| --- | --- | --- |
| 1 | - | 201.7 |
| 2 | 5.42 (dd, 7.5, 5.0) | 79.4 |
| 3 | - | 167.9 |
| 4 | 5.27 (d, 6.5) | 77.9 |
| 5 | - | 169.0 |
| 6 | 5.28 (dd, 8.5, 3.5) | 55.5 |
| 7 | 5.60 (qd, 6.0, 3.5) | 73.2 |
| 8 | - | 169.5 |
| 9 | 3.24 (dd, 7.5, 3.5) | 55.2 |
| 10 | 1.83, 1.65 (m) | 42.0 |
| 11 | 1.61 | 24.7 |
| 12 | 0.944 (d, 7.0) | 22.9 |
| 13 | 0.936 (d, 7.0) | 22.0 |
| 14 | 2.03 (m) | 37.1 |
| 15 | 1.56, 1.32 (m) | 24.8 |
| 16 | 0.94 (t, 7.5) | 15.1 |
| 17 | 0.99 (d, 6.5) | 11.1 |
| 18 | - | 170.4 |
| 19 | - | 112.9 |
| 20 | - | 150.7 |
| 21 | - | 127.6 |
| 22 | 8.58 (dd, 8.0, 1.0) | 125.1 |
| 23 | 6.96 (t, 8.0) | 119.3 |
| 24 | 7.32 (dd, 8.0, 1.0) | 120.3 |
| 25 | 8.52 (d, 1.5) | 159.1 |
| 26 | 1.38 (d, 7.0) | 16.1 |
| 27 | 1.91,1.66 (m) | 27.8 |
| 28 | 1.27 (m) | 27.0 |
| 29 | 1.28 (m) | 29.1 |
| 30 | 1.27 (m) | 31.7 |
| 31 | 1.26 (m) | 22.7 |
| 32 | 0.86 (t, 7.0) | 14.2 |
| 6-NH | 7.07 (d, 8.5) | - |
| 20-OH | 12.63 (brs) | - |
| 21-NH | 7.91 (brs) | - |

**Supplementary Table 10.** ^1^H NMR data of **7b, 7d, 7e**

|  | **7b** | **7d** | **7e** |
| --- | --- | --- | --- |
| Position | ^1^H (multiplet, J value [Hz]) | ^1^H (multiplet, J value [Hz]) | ^1^H (multiplet, J value [Hz]) |
| 2 | 5.43 (dd, 7.5, 4.5) | 5.43 (dd, 8.0, 4.5) | 5.42 (dd, 7.5, 5.0) |
| 4 | 5.45 (dd, 10.0, 4.0) | 5.45 (dd, 10.0, 4.0) | 5.27 (d, 6.5) |
| 6 | 5.26 (dd, 8.5, 3.5) | 5.26 (dd, 8.5, 3.5) | 5.28 (dd, 8.5, 4.0) |
| 7 | 5.61 (qd, 6.5, 3.5) | 5.61 (qd, 6.0, 3.5) | 5.60 (qd, 6.5, 3.5) |
| 9 | 3.25 (t, 7.0) | 3.22 (t, 7.0) | 3.24 (t, 7.0) |
| 10 | 1.93, 1.70 (m) | 1.93, 1.70 (m) | 1.91, 1.69 (m) |
| 11 | 1.62 (m) | 1.62 (m) | 1.62 (m) |
| 12 | 0.95 (d, 6.5) | 0.94 (d, 6.5) | 0.95 (d, 6.0) |
| 13 | 0.93 (d, 6.5) | 0.92 (d, 6.5) | 0.93 (d, 6.0) |
| 14 | 1.83 (m) | 1.83 (m) | 2.03 (m) |
| 15 | 1.72 (m) | 1.75 (m) | 1.56, 1.32 (m) |
| 16 | 1.00 (d, 6.5) | 1.00 (d, 6.5) | 0.94 (m) |
| 17 | 0.95 (d, 6.5) | 0.94 (d, 6.5) | 0.99 (d, 7.0) |
| 22 | 8.58 (d, 8.5) | 8.58 (d, 8.0) | 8.58 (d, 8.0) |
| 23 | 6.96 (t, 8.0) | 6.96 (t, 8.5) | 6.96 (t, 8.0) |
| 24 | 7.32 (d, 8.5) | 7.32 (d, 8.0) | 7.31 (d, 7.5) |
| 25 | 8.52 (d, 1.0) | 8.52 (d, 1.5) | 8.52 (d, 1.0) |
| 26 | 1.39 (d, 6.5) | 1.39 (d, 7.0) | 1.38 (d, 6.5) |
| 27 | 1.63 (m) | 1.95,1.68 (m) | 1.90, 1.70 (m) |
| 28 | 1.26-1.28 (m) | 1.14 (m) | 1.26-1.28 (m) |
| 29 | 1.26-1.28 (m) | 1.58 (m) | 1.26-1.28 (m) |
| 30 | 0.88 (t, 7.5) | 0.88 (d, 7.0) | 1.29 (m) |
| 31 | - | 0.87 (d, 7.0) | 0.88 (t, 7.0) |
| 6-NH | 7.08 (d, 8.5) | 7.08 (d, 8.5) | 7.07 (d, 8.0) |
| 20-OH | 12.4 (brs) | 12.4 (brs) | 12.5 (brs) |
| 21-NH | 7.91 (brs) | 7.91 (brs) | 7.91 (brs) |
